# The Effectiveness of a Four-Week Online Mindfulness Training Course on Individual Mindfulness Skills and Personal Perception of Stress in Company Employees Working from Home

**DOI:** 10.3390/ijerph192416422

**Published:** 2022-12-07

**Authors:** Madiha Rana, Lotte Bock, Erik Riedel, Henriette L. Moellmann, Lara Schorn, Majeed Rana

**Affiliations:** 1 Department of Psychology, European University of Applied Sciences for Distance Learning Hamburg, Doberaner Weg 20, 22143 Hamburg, Germany; 2 Department of Oral, Maxillo- and Plastic Facial Surgery, Heinrich-Heine-University, Moorenstr. 5, 40225 Duesseldorf, Germany

**Keywords:** working from home (WFH), home office, homeworkers, mindfulness, stress, workplace

## Abstract

Working from home comes with many benefits. However, employees are often exposed to various stressors when working outside of the traditional workplace environment. The subjective experience of these stressors is related to one’s perception of the situation and the perceived resources available. As working from home has become the new normal for many during the COVID-19 pandemic, it is in the interest of companies to provide employees with tools to cope with these stressors. One such tool is online mindfulness training. This study investigates how a four-week online mindfulness training influences an individual’s mindfulness skills, subjective perception, and processing of stressors. Forty participants working from home at the time of the study were examined in a pre-test using a pre-post design in which the experimental group participated in a four-week online training course in mindfulness. Since the results showed a significantly reduced subjective perception of stress in the experimental group after mindfulness training, a long-term study was conducted including 40 additional participants. The study revealed a training success of at least three months. It thus introduces new possibilities for effective stress management in all workplace settings.

## 1. Introduction

At the beginning of the COVID-19 pandemic in 2020, many companies were suddenly facing the challenge of enabling their employees to work from home. Both employers and employees were forced to adapt to this new situation at short notice. The rising number of employees working from home inspired several studies discussing the advantages and disadvantages of working from home. On the one hand, working from home was found to improve work–life balance, to increase efficiency and to offer a greater overall control of workflow [1]. It further saves travel time and expenses and offers an opportunity to organize the working process independently [2]. On the other hand, studies revealed that a high weekly amount of working hours from home is associated with greater stress-related symptoms and negatively influences job satisfaction [3]. Perceived stress cannot be entirely based on enforcements during the COVID-19 pandemic. Prior to the pandemic, the importance of several stress factors caused by changes in the workplace situation had already been defined. These factors are work intensification due to the combination of extra work and staff reductions, acceleration of time pressure due to strategies such as effectiveness and optimization targets, dissolution of boundaries due to a lack of separation between work and private life, informatization due to accessibility and multitasking, and demands on adaptability due to permanent changes [4,5].

The new workplace situation caused by the COVID-19 pandemic added new challenges to the existing ones in terms of family life, work-life balance (disengagement), and lack of social contact with colleagues [6]. In order to reduce stress in employees working from home, health insurance companies recommend mindfulness programs. The idea to use mindfulness to cope with stress is supported by recent research on the effect of mindfulness in a working environment [7,8].

Since the 1900s, mindfulness has been a well-established stress management method [9,10,11,12,13]. It is defined as the ability to be fully aware of the present moment without judgement [14]. Bishop et al. [15] introduced a two-component model including the ability to self-regulate one’s attention and the ability to perform a specific orientation. In the context of self-regulation, one should become aware of one’s own changing thoughts, feelings, and sensations without judging and without reacting. The orientation involves an attitude of curiosity, openness, and acceptance towards one’s own experiences in the present moment [15]. Mindfulness training is often delivered in the form of a standardized eight-week mindfulness-based stress reduction (MBSR) training. Each week includes eight sessions lasting between 2.5 and 3.5 h. In addition, there is a full-day training to deepen the experience of exercises and meditations [16]. It consists of formal and informal exercises. Formal exercises are relaxation exercises such as yoga or meditation which are supposed to be exercised for 45–60 min per day. The informal exercises are instructions and incentives for mindful behavior. These can be exercises such as mindful eating, mindful listening, or mindful driving. Additionally, each session contains group discussions for sharing experiences and self-reflection.

The benefits of mindfulness training in the workplace have gained attention in recent research. It has been demonstrated to positively affect stress, anxiety, psychological distress [17], and depression [18]. A mindfulness training seems to improve employees’ mental health, compassion and empathy, well-being, and performance [18,19,20]. It further anticipates burnout [18,21] with less emotional exhaustion and more job satisfaction [22]. Moreover, it has been suggested that a mindfulness and self-compassion-based intervention (MSCBI) may even be more effective than regular psychoeducational interventions in treating work-related stress and burnout [23]. Previous studies, however, were often not fully validated due to a lack of control groups. 

Most research on the impact of mindfulness training is focused on courses led by trainers and based on the eight-week curriculum of the MBSR program. This, however, does not resemble the recent changes in the work situation, exacerbated by the COVID-19 pandemic, where trainings had to be available online and self-administered due to restrictions. Therefore, the need for stress-reducing interventions that can be taught remotely increased. Research on mindfulness training as mentioned above often refers to the classic Kabat-Zinn eight-week online course with the recommendation of 45–60 min of daily practice at home [24,25,26]. An eight-week online mindfulness training has been found to significantly affect depression, anxiety, well-being, workplace performance [24], stress, mood, and resilience [26]. A study by Querstrert et al. [27] revealed that even a four-week training might be sufficient. However, other studies revealed that online mindfulness trainings had a small to moderate effect on stress and mindfulness in the general population [28,29]. Sommers-Spijkerman et al.’s [30] updated meta-analysis found statistically significant moderate pre-to-post effects in terms of depression, stress, and mindfulness with only small effects on anxiety. A comprehensive overview of the above-mentioned studies can be found in Table 1. In a work environment characterized by overtime, downsizing, time pressure, delimitation, multitasking, and demands for adaptability due to constant change, it seems relevant to look for less time-consuming and cost-effective mindfulness programs that can be accessed anytime and anywhere in order to meet the demands for flexibility and adaptability. A four-week remote training with easy implementation and flexible delivery, without compromising the positive impact of mindfulness on personal perceptions of wellbeing and stress, might offer a solution. 

This study therefore aims to answer the following research question: Does a four-week self-directed online training in mindfulness have a positive short- and long-term effect on an individual’s mindfulness skills and the individual’s subjective perception, evaluation, and processing of stressors?

Various research approaches have shown an improvement in key mindfulness skills through mindfulness training in different settings. Furthermore, perceived stress level could be reduced. This is in line with the theory that stress perception depends on the subjective perception, evaluation, and processing of stressors [31,32,33] and has less to do with actual circumstances. We therefore hypothesize:

**Hypothesis** **1:** *The experimental group will report significantly improved awareness of eight core mindfulness skills (accepting and non-judgmental attitude, non-reactive decentering, awareness of relativity of thoughts, empathic understanding, awareness of inner experiences, openness to experiences, awareness of outer experiences, acting with awareness) after a four-week self-directed online mindfulness course compared to the control group.*

**Hypothesis** **2:** *The experimental group will report significantly fewer stress symptoms (worry, less tension, more joy and more positive evaluation of work demands) after four weeks of self-directed online mindfulness training compared to the control group.*

**Hypothesis** **3:** *The experimental group will report significantly improved awareness of eight core mindfulness skills (accepting and non-judgmental attitude, non-reactive decentering, awareness of relativity of thoughts, empathic understanding, awareness of inner experiences, openness to experiences, awareness of outer experiences, acting with mindfulness) three months after completing four weeks of self-directed online mindfulness training compared to the control group.*

**Hypothesis** **4:** *The experimental group will report significantly fewer stress symptoms (worry and tension, increased happiness and positive appraisal of work demands) three months after completing four weeks of self-directed online mindfulness training compared to the control group.*

## 2. Material and Methods

This study was approved by our institution’s ethics review board (EKEFH01/21). In-formed written consent was obtained from each participant. 

### 2.1. Procedure and Participants

This study consists of a randomized controlled trial with two samples which will be referred to as sample 1 and sample 2 in the following. 

Sample 1 was collected during a pre-study and had two assessment periods: baseline and post-intervention. The measurement points for sample 1 was from April 2021 to May 2021 

Sample 2 was collected during a follow-up study and had three assessment periods baseline, post-intervention, and a three months follow-up. Measurement points for sample 2 were August, September and December 2021. 

For each sample, 40 participants were recruited via social media channels (Facebook, Instagram, LinkedIn and Xing) and randomly assigned to an experimental group and a wait list control group. The requirement for participation was that the respondents at baseline assessment were primarily (more than half the work hours) working from home and that the participants were willing to spend approximately 20 min a day practicing mindfulness during the time of the course. All participants were German employees. For a priori calculation of the necessary sample size, the three central influencing variables: significance level, test strength and effect size were determined using the software G*Power (Heinrich-Heine University, Düsseldorf, Germany) [34]. With a significance level of 0.05, a test strength of 95% and a mean effect size f = 0.25, η²p = 0.06, for sample 1 a sample size of 36 persons were required in order to calculate an ANOVA for 2 groups and 2 repeated measures (baseline and post-intervention) and for sample 2 a sample size of 44 persons were required in order to calculate an ANOVA for 2 groups and 3 repeated measures (baseline, post-intervention; and 3-month follow-up).

In sample 1, 33 females and 7 males participated. 17 females and 4 males were randomly assigned to the experimental group (n = 21) and 16 females and 3 males were assigned to the wait list control group (n = 19) (Table 1). Two participants were younger than 30 (5%), 7 participants were 30 to 39 years old (29%), the majority (n = 20) were 40 to 49 and 12 participants were 50 years and older (29%). Sixty-eight percent of the sample worked full time (n = 28), and 13 participants worked part time or were self-employed (32%). Twenty-three participants (56%) took care of children while working in the home office. One third of the sample (n = 12) had no previous experience with methods such as yoga, meditation or PMR, and 42.5% (n = 17) had experience with yoga and/or meditation. The remaining participants (n = 11) had experienced a mix of yoga, meditation, PMR, Thai-Chi and autogenous training. Reliability of scales and sub-scales was very good: For PSQ (T0) = 0.930 (subscales 0.842–0.742) and for CH (T0) = 0.921 (subscales 0.873–0.640).

In sample 2, 19 females and 2 males (n = 21) were randomly assigned to the ex-perimental group and 15 females, and 4 males (n = 19) were assigned to the wait list control group (Table 1). Ten participants were younger than 30 (25%), 8 participants were 30 to 39 years old (20%), the majority (n = 17) were 40 to 49 and 5 participants were 50 years and older (12.5%). Twenty-five participants of the sample worked full time (62.5%), 7 participants worked part time or were self-employed (35%). One participant was in a short contract. 

In sample 1, 18 participants in the experimental group and all of the control group worked mostly at home. Four-weeks after the webinar, 17 of the experimental group and 18 of the control group still worked at home. Sixteen of the experimental group and 14 in the control group took care of children while working from home. Twenty-seven participants were working from home voluntarily, of whom 15 were in the experimental group. One third of the sample (n = 12) had no previous experience with methods such as yoga, meditation or PMR, and 42.5% (n = 17) had experience with yoga and/or meditation. The remaining participants (n = 11) had experienced a mix of yoga, meditation, PMR, Thai-Chi and autogenous training. 

The proportion of female and male in sample one and two is given in the following table (Table 2):

### 2.2. Online Mindfulness Training 

The self-led online mindfulness training examined in the two samples was designed specifically for this study. The four-week course contained twenty-eight units and an additional introductory session. Each morning between 6:00 and 7:00 a.m. participants were automatically contacted via email. Each daily email contained three videos: one video with a mindfulness prompt in form of an informal mindfulness exercise, one video with office yoga exercises, and one video with a meditation to complement the formal exercises. The three daily components had a duration of approximately 20 min. Units could be practiced contiguously or spread throughout the day. The introductory unit contained a video on the overall structure of the training, a general video about mindfulness, and instructions on how to successfully complete the training (the program protocol can be found in the supplementary material).

The 84 video units referred to the theory of the classical MBSR program and the eight subscales of the CHIME questionnaire. The 28 units of the four-week course relate to Appraisal, Emotion Scoping, Personal Resources, Beliefs, Accepting Self-Empathy, Connecting Self-Empathy, Non-Identifying Self-Empathy, Emotional Dissonance, Volatility of the VUCA World, Uncertainty of the VUCA World, and Embodiment.

### 2.3. Questionnaires

The validated German version of the CHIME questionnaire, developed by Bergomi, Tschacher and Kupper [35], was used to measure eight basic mindfulness skills (Supplementary File S1). The CHIME questionnaire is based on a comparison of eight existing mindfulness questionnaires [36,37]. In order not to favor any of the existing questionnaires, eight sub-factors and one super-factor (mindfulness) were identified. The questionnaire aims to consider all factors equally and to create items that are easily accessible to everyone [36]. Furthermore, the questionnaire aims to capture traits describing a stable character, but can also be adapted to situational events. Studies indicate that the CHIME is more comprehensive than, for example, the Five Facet Mindfulness Questionnaire (FFMQ), since it takes factors into account that the FFMQ does not [36]. In addition, the CHIME has been tested in a before/after comparison design of MBSR training. Due to these compelling factors the CHIME was considered as appropriate for the current study.

In the development and validation of the CHIME construct validity, criterion validity, and incremental validity as well as change sensitivity were all at least satisfactory in comparison to other mindfulness scales [35]. The questionnaire includes 37 items consisting of statements in the I-form. They are to be rated on a 6-point bipolar Likert scale with six answer options.

The questions are distributed across the following eight subscales: 

Accepting and non-judgmental attitude (ANA), nonreactive decentering (ND), awareness of thought’s relativity (ATR), insightful understanding (IU), awareness of internal experiences (AIE), openness to experiences (OE), awareness of external experiences (AEE), and acting with awareness (AWA) [35]. 

The goodness criteria of the CHIME was measured using Cronbach’s alpha and indicated acceptable internal consistency (α = 0.70–0.86) for the eight factors of the CHIME [36]. The sample groups were presented with the German version of the CHIME [35]. A confirmatory factor analyses has shown an acceptable model fit of the structure of the CHIME with the following eight subscales: awareness of internal experiences, awareness of external experiences, acting with awareness, accepting and non-judgmental attitude, nonreactive decentering, openness to experiences, awareness of thought’s relativity, and insightful understanding (χ^2^ = 1534.90, df = 601, χ^2^/df = 2.55, CFI = 0.90, RMSEA = 0.057 [90% CI 0.053, 0.060], SRMR = 0.06). The CHIME Short Form (SF) has as well shown a good model fit (χ^2^ = 486.13, df = 224, χ^2^/df = 0.17, CFI = 0.95, RMSEA = 0.049 [90% CI 0.043, 0.055], SRMR = 0.05.) and the subscales of the CHIME as well as the CHIME-SF have shown high corrected correlations (rc = 0.69–0.88). Conclusively, both the CHIME and the CHIME-SF have proved to be sensitive to change [38].

The German version of the PSQ was used to measure subjective perception, appraisal, and processing of stressors [39]. The reliability was measured in regards of internal consistency is measured by Cronbach’s alpha (α = 0.80–0.86.), of the split-half reliability measured by Guttmann (r = 0.69–0.86) and Spearman-Brown (r = 0.74–0.88.) Validity findings on convergent and criterion-related validity, sensitivity to change showed that the factors worry and tension as well as the overall stress experience correlated particularly high negatively and the factor joy correlated highly positively with the mental domain of the WHO Quality of Life Questionnaire. The factor tension correlated highly negatively with the physical QoL dimension of the WHOQOL. The PSQ measures subjective perceptions of stress over the past four-weeks. The short version consists of 20 items distributed over four subscales: worry, tension, pleasure, and demands. It consists of statements to be rated on a scale of 1 to 4. These statements are consistent with the focus and goal of mindfulness training. They address how the respondent feels and thinks. Eight of 20 questions begin with “You feel...,” thus underlining the scales of the CHIME. The reliability of the PSQ questionnaire was measured using Cronbach’s alpha and showed very satisfactory internal consistency, α = 0.80–0.86 [39].

In addition to the questionnaires, the following data was collected before the training: “Are you currently employed in a home office?”, “Gender” (male, female, diverse), “Responsibilities for childcare and/or homeschooling” (yes/no), and “Employment relationship” (full time, part time, short-term). After the course, respondents were asked about the frequency of weekly practice of each of the formal and informal practices (response options were “one-two days”, “three-four days”, “five-six days”, “every day”). To assist in answering this question, participants were given an exercise log at the beginning of the course, which allowed them to track when they had completed the exercises. 

### 2.4. Statistical Analysis

To analyze the collected data we used Jamovi 2.2.5 [40]. Descriptive statistical methods were used to demonstrate the characteristics and features of the sample and collected data. Mixed factorial ANOVA with repeated measures was used to examine the differences over time represented in hypotheses 1 to 4 [41]. We controlled for outliers, tested with studentized residuals (±3) and removed them from corresponding analyses. We tested for normal distributions with the Shapiro-Wilk test of normality. The assumption of sphericity was tested with Mauchly’s test of sphericity. Stated violations of sphericity were corrected with Greenhouse–Geisser correction $\hat{\varepsilon }$ [42]. For the assumptions of homogeneity of variance, we used Levene’s Test. Test for post-hoc multiple comparisons were performed according to Tukey if the condition of variance homogeneity was met [43]. If homogeneity was violated, we performed the post-hoc tests according to Holm [44]. We calculated only planned comparisons according to our hypothesis. 

Reliability analysis was performed with Cronbach’s-α to test the internal consistency of PSQ and CHIME scores as well as for corresponding scales following Vaske et al.’s [45] recommendations for interpretation. According to our a priori power analysis we set a significant level for mixed factorial ANOVA with repeated measures and their corresponding post hoc tests on a 0.05 level. By this we ensure the power of all analysis to be at least 0.85 or higher. To be conservative in testing for sphericity for Maulchy’s test we set a significance level of 0.05.

To assess effect sizes, partial Eta-squared is reported for the mixed ANOVA with repeated measures [46]. To assess the strength of the observed significant mean differences of the Tukey HSD planned post-hoc comparisons, the following 3 statistics are reported: The mean differences and their 95% CI for the specific comparison, Cohen’s *d* as an established measure for determining effect size for group comparisons, and the Pearson Product Moment Correlation *r* converted from Cohen’s *d* to provide a standardized measure of effect size for comparison to other studies [47]. Cohen [48] has provided benchmarks to define small (η^2^ = 0.01), medium (η^2^ = 0.06), and large (η^2^ = 0.14) effects for Eta-squared. r and d are suggested to be interpreted as small ≥ 0.1, moderate ≥ 0.3 or large ≥ 0.5.

## 3. Results

### 3.1. Results of Test for Replication 

**Figure 1:**
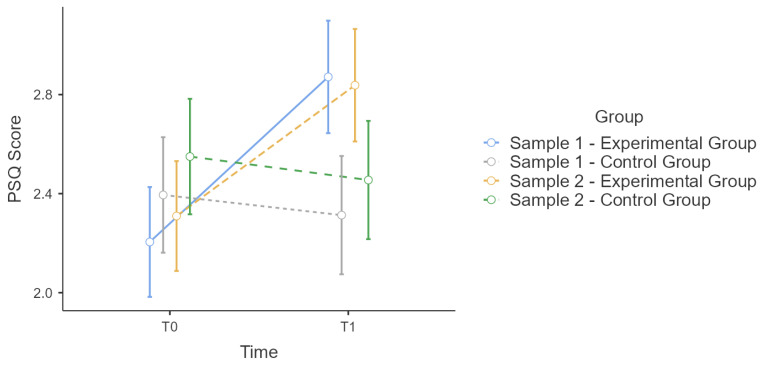
Sample 1 and 2 comparison: Estimated marginal means plot for PSQ score.

**Figure 2:**
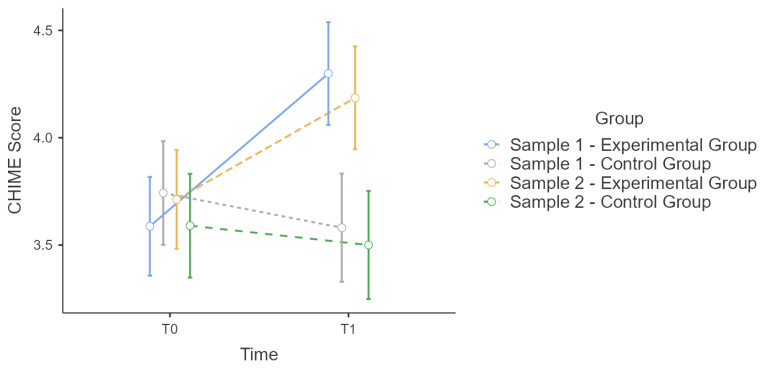
Sample 1 and 2 comparison: Estimated marginal means plot for CHIME score for.

Sample 1 and 2 correspond in age distribution and male/female ratios. We tested for effect equivalence in T0 and T1 for PSQ and CHIME with an ANOVA and group as factor. There was no significant main effect of the group in T0 for PSQ or CHIME scores. (Figure 1 and Figure 2)

Therefore, we calculated a repeated-measures ANOVA for T0 and T1 without aggregation of the experimental and control groups to show equivalence of the effect of the training. All conditions were met and there was a significant interaction effect for time * group for PSQ (F(3:76) = 8.5, *p* < 0.001, η²p = 0.25, power = 0.99) and CH (F(3:76) = 14.3, *p* < 0.001, η²p = 0.36, power = 0.99) (Table 3). Therefore, we calculated post hoc HSD accordingly to Tukey since not every group comparison is relevant. The results showed that PSQ scores (Table 4) and CHIME scores (Table 5) are consistent between samples 1 and 2. Also, the strengths of effects correspond, estimated with η²p: (η²p (PSQ-Sample 1) = 0.287, η²p (PSQ-Sample 2) = 0.210, η²p (CHIME- Sample 1) = 0.370, η²p (CHIME- Sample 2) = 0.328). By that we show that the improvement effect of the training are probably the same for sample 1 and 2.

### 3.2. Overall Results 

#### 3.2.1. PSQ—Complete

**Figure 3:**
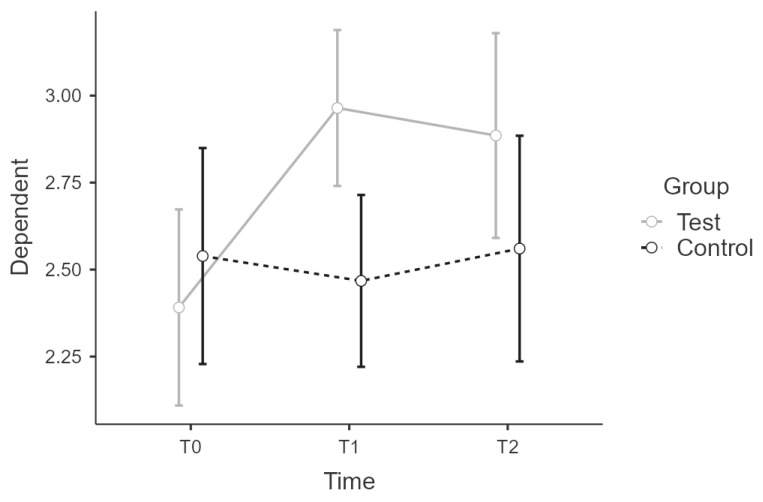
Estimated marginal means for PSQ complete score.

There was sphericity for the interaction term (W = 0.875, *p* > 0.05). The interaction between time * group on PSQ score was statistically significant, F(2:58) = 6.55, *p* = 0.003, η²p = 0.184, power = 0.99. Post hoc tests showed partial confirmation of the implicit differences between experimental and control group over time, since wbt-T2 score did not differ significantly from control-T2 score, (t(29) = 1.517, *p* = 0.656). Intervention led to significant increase of scores, t(29) = −3.587, *p* = 0.014 with a mean difference (MD) of −0.57 points, 95% CI [−0.26, −0.89], d = 1.33, r = 0.55 for t0(wbt) to t1(wbt), what is considered as large effect. There was no significant difference for t1(wbt) and t2(wbt), t(29) = 0.559, *p* = 0.993 (Figure 3). Table 4 summarizes all PSQ results across all scales.

#### 3.2.2. PSQ-JOY

Analysis showed no normality of distribution for t1 complete values. Split by group the values were normally distributed. There was sphericity for the interaction term (W = 0.954, *p* > 0.05). The interaction between time * group on PSQ-JOY score was statistically significant, F(2:58) = 4.75, *p* = 0.012, η²p = 0.141. Post hoc tests showed partial confirmation of the implicit differences between wbt and control group over time, since wbt-T2 score did not differ significantly from control-T2 score (t(29) = 1.884, *p* = 0.431). Intervention led to significant increase of scores, t(29) = −3.183, *p* = 0.037 with a mean difference of −0.45 points, 95% CI [−0.17, −0.72], d = 1.18, r = 0.51 for t0(wbt) to t1(wbt), what is considered as a large effect. There was no significant difference for t(29) = 0.218, *p* = 1. There was no significant difference between t2(wbt) and t2(con), t(29) = 1.884, *p* = 0.431.

#### 3.2.3. PSQ-WOR

**Figure 4:**
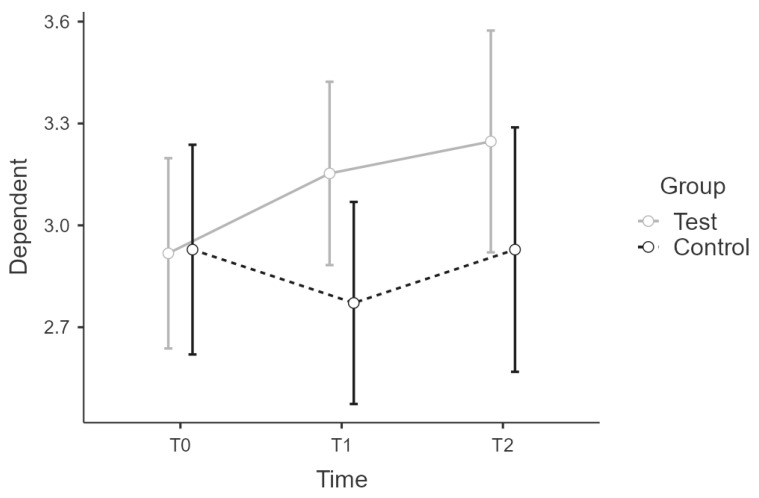
Estimated marginal means for PSQ WOR score.

Analysis showed no normality of distribution for t2 complete values. Split by group the values were normally distributed. There was no sphericity for the interaction term (W = 0.778, *p* < 0.05). The interaction between time * group on PSQ-WOR score was not statistically significant, $\hat{\varepsilon }$ = 0.82, F(1.64:47.48) = 0.417, *p* = 0.218. There were no significant main effects for time (F(1.46:47.48) = 0.455; *p* = 0.343) or group (F(1:29) = 1.215, *p* = 0.172). (Figure 4).

#### 3.2.4. PSQ-TEN

**Figure 5:**
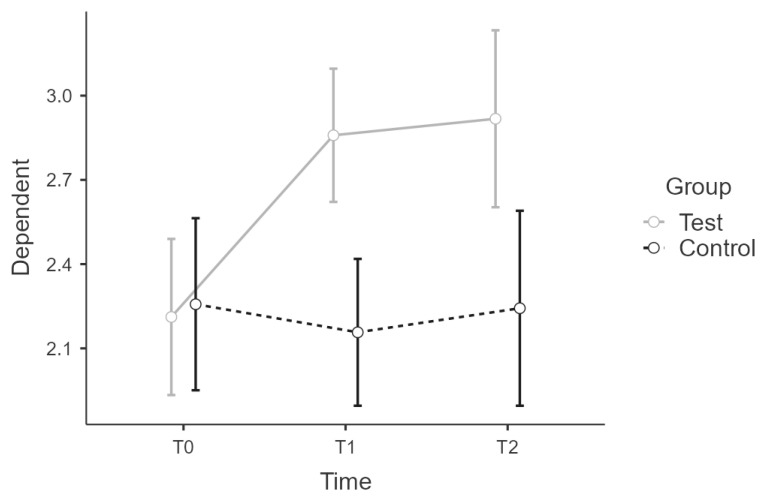
Estimated marginal means for PSQ TEN score.

Analysis showed normality of distribution and homogeneity of variance. There was sphericity for the interaction term (W = 0.961, *p* > 0.05). The interaction between time * group on PSQ-TEN Homposcore was statistically significant, F(2:58) = 6.94, *p* = 0.002, η²p= 0.193. Post hoc tests showed confirmation of the implicit differences between wbt and control group over time. Intervention led to significant increase of scores, t(29) = −4.0632, *p* = 0.004, with a mean difference of −0.65 points, 95% CI [−0.34, −0.96], d = 1.51, r = 0.60 for t0(wbt) to t1(wbt), what is considered as a large effect. There was no significant difference for t1(wbt) and t2(wbt) (t(29) = −0.365, *p* = 0.999). There was a significant difference between t2(wbt) and t2(con) (t(29) = 2.943, *p* = 0.063), d = 1.51, r = 0.60, what is considered as a large effect (Figure 5).

#### 3.2.5. PSQ-DEM

**Figure 6:**
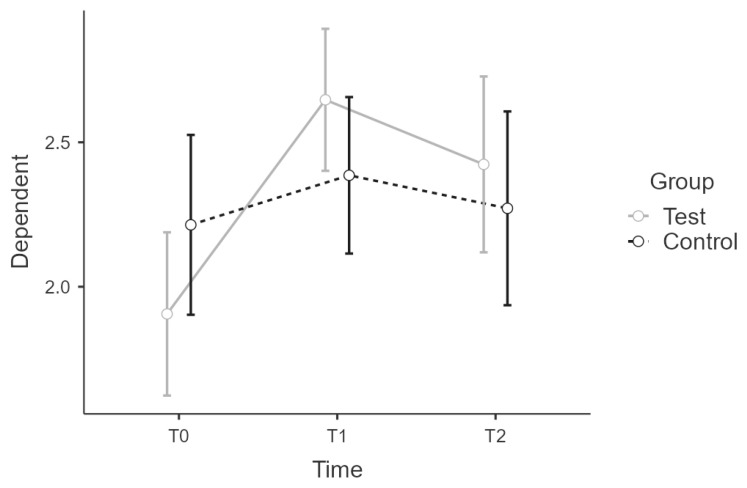
Estimated marginal means for PSQ DEM score.

Analysis showed no normality of distribution for t1 complete values. Split by group the values were normally distributed. There was sphericity for the interaction term (W = 0.957, *p* > 0.05). The interaction between time * group on PSQ-DEM score was statistically significant, F(2:58) = 4.67, *p* = 0.013, η²p= 0.139. Post hoc tests showed partial confirmation of the implicit differences between wbt and control group over time. Intervention led to significant increase of scores, t(29) = −6.146, *p* ≤ 0.001, with a mean difference of −0.74 points, 95% CI [−0.5, −0.98], d = 2.28, r = 0.75 for t0(wbt) to t1(wbt), what is considered as a large effect. There was no significant difference for t1(wbt) and t2(wbt) (t(29) = 1.547, *p* = 0.638). There was no significant difference between t1(wbt) and t1(con) (t(29) = 1.461, *p* = 0.69) as well between t2(wbt) and t2(con) (t(29) = 0.687, *p* = 0.982) (Figure 6).

#### 3.2.6. CH—Complete

**Figure 7:**
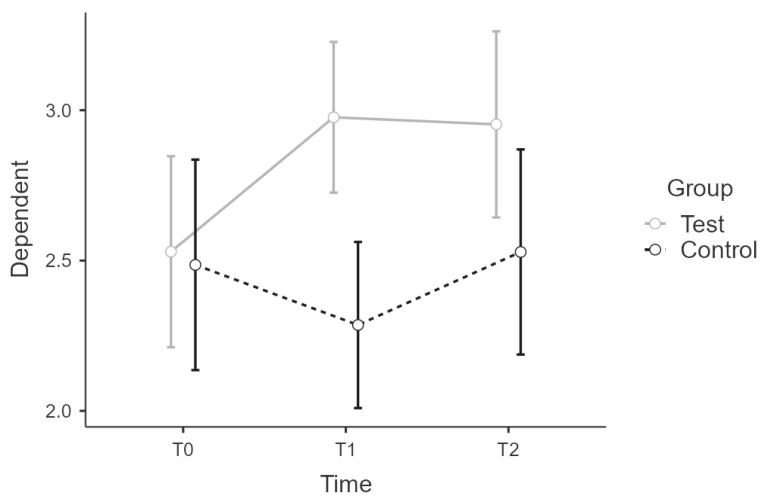
Estimated marginal means for CH complete score.

Analysis showed normality of distribution and a violation of homogeneity for t1 and t2. There was sphericity for the interaction term (W = 0.969, *p* > 0.1). There was a statistically significant interaction between time * group on CH score, F(2:58) = 10.73, *p* ≤ 0.001, η²p = 0.27, power = 0.99. Post hoc tests confirmed implicit differences between wbt and control group over time. Intervention led to significant increase of scores (t(29) = −4.798, *p* ≤ 0.001) with a mean difference of −0.49 points, 95% CI [−0.29, −0.69], d = 1.78, r = 0.67 for t0(wbt) to t1(wbt), what is considered as a large effect. There was no significant difference for t1(wbt) and t2(wbt) (t(29) = −0.721, *p* = 0.978). There was a significant difference be-tween t2(wbt) and t2(con) t(29) = 3.894, *p* = 0.006 with a mean difference of 0.94 points, 95% CI [1.42, 0.47], d = 1.45, r = 0.59, what is considered as a large effect (Figure 7). Supplementary File S2 summarizes all CH results across all scales.

#### 3.2.7. CH ANA

**Figure 8:**
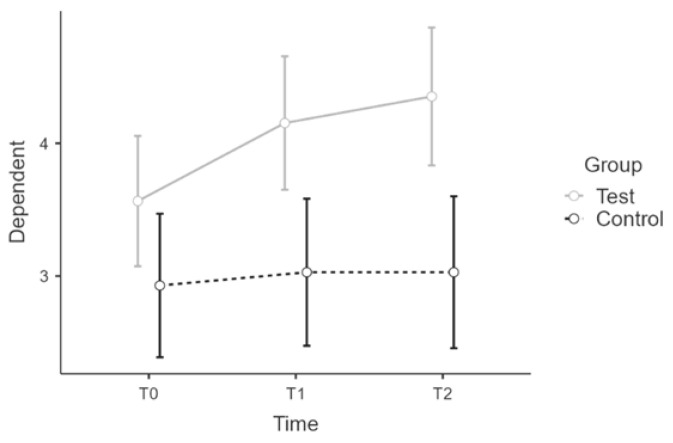
Estimated marginal means for CH ANA score.

Analysis showed normality of distribution and homogeneity of variance. There was sphericity for the interaction term (W = 0.890, *p* > 0.1). There was a statistically significant interaction between time * group on CH ANA score, F(2:58) = 3.48, *p* = 0.037, η²p = 0.107. Post hoc tests showed confirmation of the implicit differences between wbt and control group over time. Intervention led to significant increase of scores (t(29) = −3.05, *p* = 0.05) with a mean difference of 1 −0.59 points, 95% CI [−0.21, −0.97], d = 1.13, r = 0.49 for t0(wbt) to t1(wbt), what is considered as a large effect. There was no significant difference for t1(wbt) and t2(wbt) (t(29) = −1.018, *p* = 0.908). There was a significant difference between t2(wbt) and t2(con) (t(29) = 3.505, *p* = 0.017) with a mean difference of 1.32 points, 95% CI [2.06, 0.58], d = 1.30, r = 0.55, what is considered as a large effect (Figure 8).

#### 3.2.8. CH AWA

**Figure 9:**
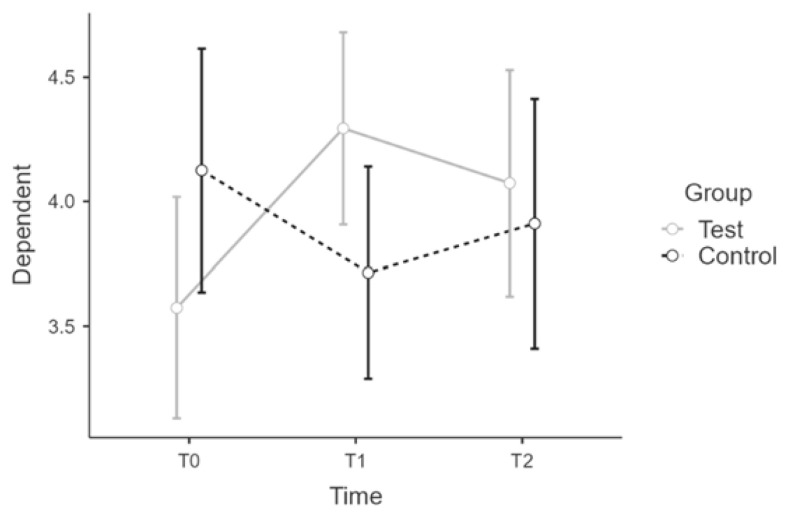
Estimated marginal means for CH AWA score.

Analysis showed normality of distribution and homogeneity of variance. There was sphericity for the interaction term (W = 0.967, *p* > 0.1). There was a statistically significant interaction between time * group on CH AWA score, F(2:58) = 6.683, *p* = 0.002, η²p = 0.187. Post hoc tests showed partial confirmation of the implicit differences between wbt and control group over time, since t2(wbt) score did not differ significantly from t2(von) score (t(29) = 0.492, *p* = 0.996) and t1(wbt) did not differ significantly from t1(con), t(29) = 2.06, *p* = 0.335. Intervention led to significant increase of scores (t(29) = −3.165, *p* = 0.038 with a mean difference of −0.72 points, 95% CI [−0.27, −1.17], d = 1.18, r = 0.51 for t0(wbt) to t1(wbt), what is considered as a large effect. There was no significant difference for t1(wbt) and t2(wbt) t(29) = 1.131, *p* = 0.864 (Figure 9).

#### 3.2.9. CH IU

Analysis showed normality of distribution and homogeneity of variance. There was sphericity for the interaction term (W = 0.902, *p* > 0.1). There was a statistically significant interaction between time * group on CH IU score, F(2:58) = 2.47, *p* = 0.093, η²p = 0.079. Post hoc tests showed partial confirmation of the implicit differences between wbt and control group over time. Intervention did not lead to a significant increase of scores in t1 to t2, t(29) = −2.6675, *p* = 0.113. But there was a significant difference for t1(wbt) and t1(con) t(29) = 3.4916, *p* = 0.018 with a mean difference of 1.09 points, 95% CI [1.7, 0.48], d = 1.30, r = 0.54, what is considered as a large effect. There was a significant difference between t2(wbt) and t2(con) (t(29) = 3.1566, *p* = 0.039) with a mean difference of 0.98 points, 95% CI [1.59, 0.37], d = 1.17, r = 0.51, what is considered as a large effect.

#### 3.2.10. CH AEE

Analysis showed normality of distribution and homogeneity of variance. There was sphericity for the interaction term (W = 0.903, *p* > 0.1). There was no statistically significant interaction between time * group on CH AEE score, F(2:58) = 1.61, *p* = 0.208. But there were significant main effects for time (F(2:58) = 2.59, *p* = 0.08, η²p = 0.08) and group (F(1:29) = 6.02, *p* = 0.02, η²p = 0.172). Since the group showed a main effect we computed a one way ANOVA with repeated measures for wbt and control group. Analysis met the requirements and showed a significance of time on CH AEE scores, F(2:32) = 4.63, *p* = 0.017, η²p = 0.0.225. Post hoc test revealed a significant difference between t0 and t2 score (t(16) = −1.885, *p* = 0.017) but not between t1 and t2 (t(16) = −1.885, *p* = 0.685). For the control group there was no significant effect of time (F(2:26) = 0.078, *p* = 0.925.

#### 3.2.11. CH AIE

Analysis showed normality of distribution and homogeneity of variance. There was no sphericity for the interaction term (W = 0.808, *p* < 0.1). There was a statistically significant interaction between time * group on CH AIE score, $\hat{\varepsilon }$ = 0.96, F(1.67:48.34) = 3.33, *p* = 0.052, η²p = 0.103. Post hoc tests showed partial confirmation of the implicit differences between wbt and control group over time, since t0(wbt) score did not differ significantly from t1(wbt) score, t(29) = −2.711, *p* = 0.104. There was a significant difference for to(wbt) and t2(wbt) (t(29) = −2.919, *p* = 0.978) with a mean difference of −0.55 points, 95% CI [−0.18, −0.92]. There was a significant difference between t1(wbt) and t1(con) t(29) = 2.941, *p* = 0.064 with a mean difference of 0.86 points, 95% CI [1.43, 0.29]. Also there was a significant difference between t2(wbt) and t2(con) (t(29) = 3.067, *p* = 0.048) with a mean difference of 0.85 points, 95% CI [1.4, 0.31], d = 1.14, r = 0.49, what is considered as a medium effect.

#### 3.2.12. CH ND

Analysis showed normality of distribution and a violation of homogeneity for t1 and t2. There was sphericity for the interaction term (W = 0.995, *p* > 0.1). There was a statistically significant interaction between time * group on CH ND score, F(2:58) = 8.28, *p* ≤ 0.001, η²p = 0.222. Post hoc tests showed confirmation of the implicit differences between wbt and control group over time. Intervention led to significant increase of scores (t(29) = −3.115, *p* = 0.043) with a mean difference of −0.58 points, 95% CI [−0.21, −0.94], d = 1.16, r = 0.50, what is considered as a large effect. There was no significant difference for t1(wbt) and t2(wbt) (t(29) = −1.31, *p* = 0.777). There was a significant difference between t2(wbt) and t2(con) (t(29) = 4.769, *p* ≤ 0.001) with a mean difference of 1.4 points, 95% CI [1.98, 0.83], d = 1.77, r = 0.66, what is considered as a large effect.

#### 3.2.13. CH OE

Analysis showed normality of distribution and a violation of homogeneity for t1. There was sphericity for the interaction term (W = 0.829, *p* > 0.1). There was a statistically significant interaction between time * group on CH OE score, F(2:58) = 3.55, *p* = 0.035, η²p = 0.109. Post hoc tests showed partial confirmation of the implicit differences between wbt and control group over time, since t2(wbt) score did not differ significantly from t2(con) score, t(29) = 1.686, *p* = 0.552. But Intervention led to significant increase of scores (t(29) = −4.273, p = 0.002) with a mean difference of −0.71 points, 95% CI [−0.38, −1.03], d = 1.59, r = 0.62, what is considered as a large effect. There was no significant difference for t1(wbt) and t2(wbt) (t(29) = 0.7481, *p* = 0.974). There was no significant difference between t1(wbt) and t1(con) t(29) = 2.7318, *p* = 0.099).

#### 3.2.14. CH ATR

Analysis showed normality of distribution and a violation of homogeneity for t2. There was sphericity for the interaction term (W = 0.867, *p* > 0.1). There was a statistically significant interaction between time * group on CH ATR score, F(2:58) = 3.548, *p* = 0.035, η²p = 0.109. Post hoc tests showed partial confirmation of the implicit differences between wbt and control group over time. Intervention led to no significant increase of scores (t(29) = −1.323, *p* = 0.77). But there was significant difference between t1(wbt) and t1(con) (t(29) = 3.535, *p* = 0.016, MD = 1.11, 95% CI [1.72, 0.49], d = 1.31, r = 0.55, what is considered as a large effect ) and t2(wbt) and t2(con) (t(29) = 4.106, p = 0.004, MD = 1.29, 95% CI [1.91, 0.67], d = 1.52, r = 0.61, what is considered as a large effect

An overview of homogeneity, normality, and sphericity across all scales of the questionnaires is presented in Table 6.

Post-hoc power analyses were performed using G*Power to determine the achieved power (1-β err prob) of the interaction effects for CHIME and PSQ as given in Table 2. Power greater than 0.8 was found in each case. The correlations between repeated measures scores averaged 0.6 for CHIME and 0.38 for the PSQ questionnaire.

## 4. Discussion

This study aimed to evaluate the short-term and long-term effect of a four-week self-led online mindfulness training course on individual mindfulness skills and personal perception of stress in home office. The question was examined using a pre-post design with 80 participants primarily working from home due to the COVID-19 pandemic at the time of the study. 

First, 40 participants (sample 1) were evaluated to examine whether central mind-fulness skills and the subjective perception of stress can significantly improve after the four-week self-led online training intervention. In the next step, we examined the short- and long-term effectiveness of the mindfulness training in another 40 participants (sample 2). 

### 4.1. Short-Term Effects on Mindfulness Skills and Perception of Stress 

In line with our hypothesis 1 and 2, examining the short-term effect of the four-weeks self-led online training in sample 2, we found that mindfulness training significantly enhanced the overall central mindfulness skills as well as the overall perception of stress immediately after the four-week intervention. These findings confirmed the results of sample 1 after four-weeks of training. 

Of the eight examined mindfulness skills we found that in sample 1 four skills were significantly improved in post assessment immediately after the training. These were accepting and non-judgmental attitude (ANA), nonreactive decentering (ND), awareness of thought’s relativity (ATR) and insightful understanding (IU). Awareness of internal experiences (AIE), openness to experiences (OE), awareness of external experiences (AEE) and acting with awareness (AWA) were not significantly changed immediately after training. In sample 1 we had found seven scales to be significantly changed and only one scale, openness to experiences (OE), not to be significantly influenced. Openness to experiences refers to the ability of confronting instead of suppressing unpleasant emotions, which is part of a successful emotional regulation. Emotional regulation has been found to be strongly connected to mindfulness [12,49], but the reason for OE not significantly changing in our results, might be due to the time factor. Four-weeks might have been too short to change innate coping strategies dealing with particularly unpleasant emotions. Furthermore, dealing with inherent coping strategies might require the support of a therapist or coach. 

In sample 1, all four scales measuring the perception of stress had significantly improved after the four-week self-led training. In sample 2, we only found two scales to have changed significantly after four-weeks: joy and tension. In other words, we were not able to confirm a significant improvement in the scales worries and demands directly after the training. This mirrors the inconsistent findings on the effect of mindfulness training on the perception of stress described in literature. However, the overall perception of stress was significantly improved in both samples, confirming our second hypothesis. In a different study, Martínez-Borrás et al. [23] found a positive effect of mindfulness training in a work environment as well. However, they only investigated a certain aspect of mindfulness (being compassionate) whereas in our study mindfulness was assessed comprehensively.

The first sample was collected from April to May 2021 and the second sample from August to December 2021, so participants in the first study experienced longer days and fewer COVID-19 cases, which may have affected the level of worry. On the contrary, participants in the second sample experienced shorter days and another wave of COVID-19 cases delaying the end of the pandemic with prolonged restrictions. This may have led to greater concern. 

### 4.2. Long-Term Effects on Mindfulness Skills and Perception of Stress 

Hypothesis 3 and 4, which examined the long-term effectiveness of the four-week self-led training were partly confirmed. The long-term effect was only measured in sample 2. We found a significant difference between the overall mindfulness scores of the experimental group and the control group, but we only found a significant difference in one of four scales regarding the perception of stress three months after the training. 

The four mindfulness skills, which we had found to be significantly enhanced in the experimental group of the second sample compared to the control group, were still significantly higher than the control group in the follow-up assessment three months after the training. In addition to a non-judgmental attitude (ANA), nonreactive decentering (ND), awareness of thought’s relativity (ATR) and insightful understanding (IU) we also found that awareness of internal experiences (AIE) had significantly improved in the experimental group of the second sample in the follow-up assessment. 

These five long-term enhanced mindfulness skills are essential parts of the definition of mindfulness. According to Jon Kabat-Zinn’s theory and definition, mindfulness is a mindful state of awareness, with an intentional, non-judgmental (ANA), non-reactive (ND), non-willful directing of attention to the present moment (AIE/ATR) [14]. Bishop et al.’s [15] two additional subcomponents of mindfulness “self-regulation of attention” and “specific orientation” addresses the ability to become aware of one’s own changing thoughts (ATR), feelings, and sensations (IU), non-judgmentally (ANA) and non-reactively (ND). All of these skills were found to be improved on a long-term basis. 

Neff [50] describes the importance of an accepting, kind, empathic attitude in dealing with stress, which indicates that increased scores in ANA might lead to better stress management skills. Accepting challenging circumstances in a non-judgmental manner is seen as a key skill in reducing the secondary stress caused by worries and rumination. Studies have shown that acceptance activates frontal and parietal areas of the brain, enabling us to properly respond to a changing environment, make conscious decisions and to take cognitive control [51]. This on the other hand reduces the emotional, habitual responses of the subcortical structures of the brain, including the amygdala. When an individual experiences psychological or physical stress the amygdala activates a stress reaction through the hypothalamus and the brainstem and releases adrenaline, nor-adrenaline, and cortisol. This impairs the prefrontal cortex and the ability to regulate emotions and actions. By consciously activating the frontal cortex through acceptance the subcortical stress reaction is reduced. 

Insightful understanding (IU) and the awareness of internal experiences (AIE) furthermore confirm Ellis’ theory of rational-emotive behavior therapy (REVT) [33,52] and Lazarus and Folkman’s transactional stress model [31]. Like Lazarus, Ellis’ basic assumption is that experiences and events are initially filtered by personal perception before the individual responds emotionally and cognitively. Awareness of and insightful understanding for the internal experience are therefore paramount in order to cope with stress. Furthermore, Ellis’s and Lazarus’s theories support the importance of awareness of thought’s relativity (ATR) in order to cope with external and internal stressors. 

The scale ATR improved in the experimental groups of both samples during the mindfulness training and even continued to improve after the training was ended. Meanwhile the trajectory decreased in the control group in the post assessment and continuously decreased between the post and follow-up assessment. This decrease might be due to external factors such as decreased exposure to sunlight as the days became shorter, as well as the uncertainty regarding the outbreak of the fourth COVID-19 wave, which was forecasted for the fourth quarter of 2021. At the same time ATR continued to improve in the experimental group despite equal external circumstances. This might indicate that the participants in the experimental group continued to practice awareness of thought’s relativity after the training was ended. 

The same trajectories were seen in the scale non-reactive decentering (ND) referring to the ability to “take a step back” when tangled up in uncomfortable thoughts and feelings. The improvement seen in the experimental group in the post assessment continued to increase between the post and follow-up assessment. Meanwhile the scale values continued to drop in the control group. The explanation for the downwards trajectory in the control group might also be found in external circumstances caused by the pandemic or change of season. The upward trajectory of the experimental group might also be due to practice and getting accustomed with the mindful thinking, as could be the case in regard to ATR. 

Differences in awareness of internal experiences (AIE) was not found to be significant in our post assessment of sample 2, but in the follow-up assessment. This development might be due to the awareness of internal experience improving over time, which supports the trajectories seen in ATR and ND. 

The orientation, which Bishop et al. [15] refer to as involving an attitude of curiosity, openness, and acceptance, is covered by the scale openness to experiences (OE). We did not find significant changes on this scale in the long-term assessment either. As mentioned, this might be caused by the complexity of applying new emotional regulation strategies to cope with unpleasant emotions. A complexity which might be too hard to handle without professional help. 

Nor did we find evidence to support the hypothesis that awareness of external experiences (AEE) and acting with awareness (AWA) were significantly changed three months after the self-led online training had ended. The awareness of external experiences which contains awareness of sounds, colors and sensory inputs in the environment, were mainly addressed in the two-minute daily videos of the training. The videos with daily office yoga and the daily meditation were in comparison of an average of six to eight minutes. This might have caused the participants to focus more on the internal experiences instead of the external. 

Finally, AWA refers to a conscious behavior, which the participant actively carries out in real life. All other scales on the contrary refer to the mindset behind a potential mindful action. The fact that AWA was not significant in neither the post nor the follow-up assessment might indicate that mindfulness taught in a short time span primarily supports a certain mindset and only indirectly aims at a behavioral change. A mindful mindset might eventually lead to acting with awareness, but four-weeks might be too short in duration to consolidate and internalize a mindful mindset. 

Our findings thereby provide strong evidence that a four-week self-led online mindfulness training program can have significant long-term effects on central mindfulness skills in general and certain mindfulness skills in particular. 

We were not able to confirm hypothesis 4 surmising that the overall perceived stress level would stay significantly improved even three months after the self-led online training. In our follow-up assessment we only found the scale Tension to have remained significantly better in the experimental group compared to the control group. 

The actual stressors were not explored in the study and therefore it is not possible to make a statement on whether the participants actually felt stressed by the home office situation or by the pandemic. It can only be concluded that the perception of joy and tension were significantly improved in the experimental group directly after the intervention in both sample 1 and 2 and the perception of tension was still significantly improved after three months in sample 2. This indicates that the four-week self-led mindfulness training had a positive effect on the perception of physical tension which in line with the theory of Embodiment [53] suggests that less physical tension leads to less mental tension. This effect is most likely connected to the benefit of regular physical activity (e.g., through office yoga exercises) and regular mental relaxation (e.g., through breathing exercises and/ or meditation). 

Conclusively, our study shows that both mindfulness skills and the perception of stressors can be significantly positively altered after four-weeks of self-led online mindfulness training and that strong long-term effectiveness in core mindfulness skills can be seen even after three months. 

### 4.3. Limitations and Directions for Future Research

Eighty-five per cent of the participants were female. This is consistent with current literature stating that females are more likely to sign up for health interventions [54] but further studies should specifically investigate whether the results found here are equally applicable to men. We had deliberately not defined at what time or place the participants should carry out the exercises, since we wanted to keep the execution flexible in order to ensure low-threshold training. Thus, we were not able to control to what extent the participants adhered to their daily exercises. 

Finally, it cannot be ruled out that intervening events may have influenced subsequent scores on the CHIME and PSQ. At the time of study, the fourth wave of COVID-19 hit Germany, which might have raised additional concerns, worries and uncertainty among the participants and is likely to have had an intervening effect on perceptions. 

Previous research has generally found a moderate to strong correlation between mindfulness training and emotional resilience and satisfaction [18,22,38,55,56]. Meta-analyses have examined the effects of web-based mindfulness training [24,28,29]. They all found small to moderate effects on stress of an eight-week online instructor led mindfulness course. Our study suggests that mindfulness not only has a positive effect when taught instructor led over eight weeks with 45–60 min of daily meditation exercises but the positive effect can also be demonstrated after only four-weeks with an average of 20 min of daily exercises taught through self-led online training. Further research is essential to investigate whether these findings can be generalized for all four-week self-led online mindfulness courses. The impact of the participant’s external and personal circumstances and the impact of the trainer’s presentation and qualifications also need further research. 

In addition, further research is recommended to investigate the causality between practicing certain mindfulness skills and the perception of certain stressors. In order to do this more research has to be carried out into the perceived stress factors prior to the intervention. With such knowledge, specific mindfulness skills could be targeted in corporate, private and therapeutic settings in order to target certain stress factors. 

Another area of interest is the effectiveness of the correlation with general work satisfaction at the start of the training. It would be interesting to examine if an individual’s level of work satisfaction has an impact on how the taught mindfulness skills are perceived and applied. 

Mindfulness training consists of a combination of formal and informal exercises—inviting reflection, understanding and alternative behaviors—and exercises that relax the nervous system through breathing techniques, meditation and yoga. Further research should investigate to what extent and in what combination these two components have the highest significance on the personal stress level. Furthermore, it is also of interest to investigate the optimal length of the exercise sessions. In this study daily practice sessions averaged 20 min per day; in the classic MBSR course, 45 to 60 min a day is recommended. It would be interesting to know the minimum number of minutes of meditation and minimum number of minutes of yoga required to achieve significant effects. 

## 5. Conclusions

This study indicates that a four-week self-led online mindfulness training course is an effective stress-coping strategy which significantly affects specific mindfulness skills and the perception of certain stressors in the short and long term. The results argue well for the benefits of conducting mindfulness training in shorter periods of time and as self-led online training.

The study demonstrates that stress management in the form of self-led online mindfulness training is a valid alternative to instructor led on-site training offering a very cost-effective way to train a large number of people at the same time. 

Secondly, the results suggest that mindfulness skills can be learned in less time than previously thought. 

Thirdly, the study might help organizations to consider new, cost-effective health interventions for employees working at home or on different sites. However, an improvement in the perception of stress was not significant after three months and therefore more research needs to be carried out about the transfer of the acquired mindfulness skills into dealing with stressors of everyday life.

It is important to note that short cost-effective interventions like this should not be used to paint over institutionalized stress-causing problems inherent in the structure and traditions of an organization [57]. Nor should training be seen as an everlasting solution to eliminate stress among the employees. Organizations should ensure follow-up interventions to maintain the positive effectiveness of a self-led online mind-fulness training course and to ensure the long-term effect. 

### Practitioner Points 

Mindfulness training is an effective stress-coping strategy, which significantly affects specific mindfulness skills and the perception of certain stressors in the short and long term. 

Self-led online mindfulness training courses can improve mental health within four-weeks. 

The study helps organizations to consider new, cost-effective health interventions for employees working at home or on different sites.

## Figures and Tables

**Table 1:** Comprehensive overview of the recent literature on mindfulness training.

Author	Title	Objectives and Method	Main Findings
Bartlett et al. (2019) [17]	A systematic review and meta-analysis of workplace mindfulness training randomized controlled trials.	This meta-analytic review examines the current literature and public domains regarding the benefits of workplace mindfulness training.	The results of the review indicate beneficial effects due to the training for mindfulness, stress, anxiety, psychological distress, and for well-being and sleep.
El Morr et al. (2020) [24]	Effectiveness of an 8-Week Web-Based Mindfulness Virtual Community Intervention for University Students on Symptoms of Stress, Anxiety, and Depression: Randomized Controlled Trial	In a randomized controlled trial this study assess the effectiveness of an 8-week web-based mindfulness and cognitive behavioral therapy program. It is examined how well the programm reduces symptoms of depression, anxiety, and stress and increases mindfulness. N = 160 undergraduate students.	The study found that depression and anxiety symptoms were significantly reduced and that mindfulness scores were significant increase, but it did not find a significant effect on perceived stress.
Johnson et al. (2020) [19]	Mindfulness training in the workplace: exploring its scope and outcomes	This integrative literature review was conducted in order to compare and contrast relevant articles and information as well as create new knowledge and point out new research directions on mindfulness practices in work settings.	The review compares 28 empirical studies and concludes that mindfulness-based training is an effective intervention for organizations to improve mental health, wellbeing and performance of employees.
Kersemaekers et al. (2018) [21]	A Workplace Mindfulness Intervention May Be Associated with Improved Psychological Well-Being and Productivity. A Preliminary Field Study in a Company Setting	The study examines the feasibility and effectiveness of a Workplace Mindfulness Training in regards to burnout, psychological well-being, organizational and team climate, and performance. The constructs were measured by 425 participants up to a month before, at start of, and right at the end of a Workplace Mindfulness Training.	Comparing the intervention period with the pre-intervention period significantly greater improvements were found in terms of burnout, perceived stress, the Mindfulness Attention Awareness Scale (MAAS), and well-being.
Ma et al. (2018) [25]	Effectiveness of Online Mindfulness-Based Interventions on Psychological Distress and the Mediating Role of Emotion Regulation	The study examines the effects of eight weeks online mindfulness-based programs in terms of psychological distress (depression and anxiety). 76 participants were deived ramdomely into four groups: group mindfulness-based intervention (GMBI), self-direct mindfulness-based intervention (SDMBI), discussion group (DG) and a control group (BCG) who all completed a pre- and post-test.	Significant pre- and post-test differences in terms of mindfulness, emotion regulation difficulties, and psychological distress were seen in the GMBI and SDMBI group.
Nadler et al. (2020) [26]	Online Mindfulness Training Increases Well-Being, Trait Emotional Intelligence, and Workplace Competency Ratings: A Randomized Waitlist-Controlled Trial	The study examines the effectiveness of an online 8-week mindfulness-based training program through a randomized waitlist-controlled trial in a sample of 102 adults employed fulltime at a Fortune 100 company.	The intervention group showed statistically significant decreases in stress and negative mood and significant increases in resilience, and positive mood. The study argues that an online-based mindfulness training program enhances well-being, self-perceptions of emotional intelligence, and workplace performance.
Pérez-Fuentes (2020) [20]	Mindfulness for Preventing Psychosocial Risks in the Workplace: A Systematic Review and Meta-Analysis	The Review and Meta-Analysis examines the findings on the efficacy of MBIs on psychological variables in the workplace published from 2009 to 2019. Out of 468 articles 24 studies were included in the systematic review and meta-analysis.	The result of the meta-analysis argues that organizational interventions in mindfulness positively influence psychological variables related to employee health and wellbeing, and recommends further research to confirm this finding.
Querstret et al. 2018) [27]	The Effects of an Online Mindfulness Intervention on Perceived Stress, Depression and Anxiety in a Non-clinical Sample: A Randomised Waitlist Control Trial	The study examines the effects of an 4-week online mindfulness intervention in terms of perceived stress, depression and anxiety, as well as different facets of mindfulness. 118 adults were randomised to either an intervention (INT) or waitlist control (WLC) group. Participants were encouraged to complete the course within 4 weeks. The constructs were assessed at baseline, post-treatment, 3- and 6-month after the treatment.	Participants of the mindfulness-based intervention reported significantly lower levels of perceived stress, anxiety and depression compared with waitlist control participants. The effects were maintained at follow-up.
Sommers-Spijkerman et al. (2021) [29]	New Evidence in the Booming Field of Online Mindfulness: An Updated Meta-analysis of Randomized Controlled Trials	The meta-analysis conducts a systematic literature search up to December 2020 on the effects of online mindfulness based interventions on mental health and the potential moderators of these effects. The analysis includes 97 trials. Pre-to-post and pre-to-follow-up were calculated in terms of depression, anxiety, stress, well-being, and mindfulness using a random effects model.	The meta-analysis found a statistically significant moderate pre-to-post effects on depression stress, and mindfulness and small effects on anxiety. For well-being, a significant small effect was found only when omitting outliers or low-quality studies. Significant but small follow-up effects were found for depression and anxiety.
Zhang et al. (2020) [28]	A Meta-Analysis: Internet mindfulness-based interventions for stress management in the general population	The meta-analysis conducts a systematic literature search up to April 2019 on the effects of internet mindfulness based interventions for stress reduction in the general population. 16 Studies were included in the analysis.	The meta-analysis indicated that internet mindfulness based interventions had small to moderate effects on stress and mindfulness compared with the control group.

**Table 2:** Sample Survey 1 and 2.

	Sample 1		Sample 2	
	EG	CG	EG	CG
Female	17	16	19	15
Male	4	3	2	4
Sample size	21	19	21	19
Age 18–29	2	0	5	4
Age 30–39	2	4	4	4
Age 40–49	9	11	10	7
Age ≥ 50	6	6	2	3
In Home Office T0	19	18	18	19
In Home Office T1	20	16	17	18
In Home Office T2	n.a.	n.a.	15	13
Employed full-time	13	15	13	12
Employed, half-time	4	3	4	3
Self-employed	n.a.	n.a.	4	3
Short-time-working	3	3	0	1
Care for Children/Home Schooling	14		8	8
No prior Experience with Yoga, Meditation or PMR	8	5	4	8
Experience with Yoga, Meditation or PMR	13	14	17	10
Regular practice within 12 Month	4	5	5	5

EG: experimental Group, CG: control Group.

**Table 3:** Repeated Measures ANOVA to test effect equivalence for PSQ and CHIME.

		Sum of Squares	df	Mean Square	F	*p*		η²p
CHIME	Time * Group	5.47	3	1.82	14.30	0.00	***	0.36
	Residual	9.71	76	0.13				
PSQ	Time * Group	4.79	3	1.60	8.54	0.00	***	0.25
	Residual	14.21	76	0.19				

Note. Type 3 Sums of Squares, df = degrees of freedom, F = F-statistics, *p* = level of significance: * significant, ** highly significant, *** very highly significant, η²p = partial eta-squared.

**Table 4:** Sample 1 and 2 post hoc comparison for PSQ score to test effect equivalence.

						95% CI								
					MD	Upper	Lower	SE	df	t	p_tukey_		d	r
T0	E-S1	=	T0	C-S1	−0.19	0.13	−0.51	0.16	76.00	−1.18	0.936			
T0	E-S2	=	T0	C-S2	−0.24	0.08	−0.56	0.16	76.00	−1.49	0.812			
T0	E-S1	=	T0	E-S2	−0.10	0.20	−0.41	0.16	76.00	−0.67	0.998			
T0	C-S1	=	T0	C-S2	−0.16	0.17	−0.48	0.17	76.00	−0.94	0.981			
T1	E-S1	>	T1	C-S1	0.56	0.88	0.23	0.17	76.00	3.37	0.03	**	0.77	0.36
T1	E-S2	>	T1	C-S2	0.38	0.71	0.06	0.17	76.00	2.31	0.30	^†^		
T1	E-S1	=	T1	E-S2	0.03	0.35	−0.28	0.16	76.00	0.21	1.00			
T1	C-S1	=	T1	C-S2	−0.14	0.19	−0.48	0.17	76.00	−0.84	0.99			
T0	E-S1	<	T1	E-S1	−0.67	−0.41	−0.93	0.13	76.00	−5.00	0.001	***	1.15	0.50
T0	E-S2	<	T1	E-S2	−0.53	−0.27	−0.79	0.13	76.00	−3.96	0.004	***	0.91	0.41
T0	C-S1	=	T1	C-S1	0.08	0.36	−0.19	0.14	76.00	0.58	1.00			
T0	C-S2	=	T1	C-S2	0.09	0.37	−0.18	0.14	76.00	0.68	0.997			

Note. MD = mean difference, CI = confidence intervals, SE = standard error, df = degrees of freedom, t = value of t-statistics, *p* = level of significance: * significant, ** highly significant, *** very highly significant, d = Cronbach’s Delta, r = Pearson Product Moment Correlation, † = not as expected.

**Table 5:** Sample 1 and 2 post hoc comparison for CHIME score to test effect equivalence.

						95% CI								
					MD	Upper	Lower	SE	df	t	p_tukey_		d	r
T0	E-S1	=	T0	C-S1	−0.16	0.17	−0.48	0.17	76.00	−0.93	0.983			
T0	E-S2	=	T0	C-S2	0.12	0.45	−0.21	0.17	76.00	0.73	0.996			
T0	E-S1	=	T0	E-S2	−0.13	0.19	−0.44	0.16	76.00	−0.77	0.994			
T0	C-S1	=	T0	C-S2	0.15	0.49	−0.18	0.17	76.00	0.89	0.986			
T1	E-S1	>	T1	C-S1	0.72	1.06	0.37	0.18	76.00	4.11	0.002	**	0.94	0.43
T1	E-S2	>	T1	C-S2	0.69	1.03	0.34	0.18	76.00	3.93	0.004	**	0.90	0.41
T1	E-S1	=	T1	E-S2	0.11	0.45	−0.22	0.17	76.00	0.66	0.998			
T1	C-S1	=	T1	C-S2	0.08	0.43	−0.27	0.18	76.00	0.45	1.000			
T0	E-S1	<	T1	E-S1	−0.71	−0.50	−0.93	0.11	76.00	−6.45	0.001	***	1.48	0.59
T0	E-S2	<	T1	E-S2	−0.47	−0.26	−0.69	0.11	76.00	−4.29	0.001	***	0.98	0.44
T0	C-S1	=	T1	C-S1	0.16	0.39	−0.07	0.12	76.00	1.39	0.858			
T0	C-S2	=	T1	C-S2	0.09	0.32	−0.14	0.12	76.00	0.78	0.994			

Note. MD = mean difference, CI = confidence intervals, SE = standard error, df = degrees of freedom, t = value of t-statistics, *p* = level of significance: * significant, ** highly significant, *** very highly significant, d = Cronbach’s Delta, r = Pearson Product Moment Correlation.

**Table 6:** Test results for requirements of two-way mixed ANOVA with repeated measures.

	Spherificity			Normality			Spherificity
	T0	T1	T2	T0	T1	T2	met
PSQ	met	met	met	met	met	met	yes
JOY	met	met	met	met	**not**	met	yes
WOR	met	met	met	met	met	**not**	no
TEN	met	met	met	met	met	met	yes
DEM	met	met	met	met	**not**	met	yes
CHIME	**not**	**not**	met	met	**not**	met	yes
ANA	met	met	met	**not**	met	met	yes
AWA	met	met	met	met	met	met	yes
INU	met	met	met	met	met	met	yes
AEE	met	met	met	met	met	met	yes
AIE	met	met	met	met	met	met	no
NRD	**not**	**not**	met	met	met	met	yes
OEX	**not**	met	met	met	met	met	yes
ATR	met	**not**	met	met	met	met	yes

Note. Any conditions that are not met are shown in bold.

## Data Availability

The datasets used and/or analysed during the study are available from the corresponding author on reasonable request.

## Supplementary Materials

The following supporting information can be downloaded at: https://www.mdpi.com/article/10.3390/ijerph192416422/s1.

## References

[1] Ipsen C., van Veldhoven M., Kirchner K., Hansen J.P. (2021). Six Key Advantages and Disadvantages of Working from Home in Europe during COVID-19. Int. J. Environ. Res. Public Health.

[2] Simenenko O., Lentjushenkova O. Advantages and disadvantages of distance working. Proceedings of the International Conference at Brno University of Technology, Faculty of Business and Management.

[3] Niebuhr F., Borle P., Börner-Zobel F., Voelter-Mahlknecht S. (2022). Healthy and Happy Working from Home? Effects of Working from Home on Employee Health and Job Satisfaction. Int. J. Environ. Res. Public Health.

[4] Treier M. (2019). Gefährdungsbeurteilung Psychischer Belastungen: Begründung, Instrumente, Umsetzung (Essentials).

[5] Praeg C.-P., Bauer W. (2016). Vom Zukunftstrend zum Arbeitsalltag 4.0: Die Zukunft der Arbeit im Spannungsfeld von Work-Life-Separation und Work-Life-Integration. HR-Exzellenz.

[6] Herrmann M., Frey Cordes R. (2020). Homeoffice Im Zeichen Der Pandemie: Neue Perspektiven Für Wissenschaft UND Praxis?.

[7] Spies R. (2020). Gut durch die Krise kommen. Bankmagazin.

[8] Toniolo-Barrios M., Pitt L. (2021). Mindfulness and the challenges of working from home in times of crisis. Bus. Horiz..

[9] Grossman P., Niemann L., Schmidt S., Walach H. (2004). Mindfulness-based stress reduction and health benefits. J. Psychosom. Res..

[10] Hofmann S.G., Sawyer A.T., Witt A.A., Oh D. (2010). The effect of mindfulness-based therapy on anxiety and depression: A meta-analytic review. J. Consult. Clin. Psychol..

[11] Shigaki C.L., Glass B., Schopp L.H. (2006). Mindfulness-Based Stress Reduction in Medical Settings. J. Clin. Psychol. Med. Settings.

[12] Guendelman S., Medeiros S., Rampes H. (2017). Mindfulness and Emotion Regulation: Insights from Neurobiological, Psychological, and Clinical Studies. Front. Psychol..

[13] Baer R. (2016). Assessment of mindfulness and closely related constructs: Introduction to the special issue. Psychol. Assess..

[14] Kabat-Zinn J. Mindfulness-Based Interventions in Context: Past, Present, and Future. https://institutpsychoneuro.com/wp-content/uploads/2015/09/Kabat-Zinn-2003.pdf.

[15] Bishop S.R., Lau M., Shapiro S., Carlson L., Anderson N.D., Carmody J., Segal Z.V., Abbey S., Speca M., Velting D. (2004). Mindfulness: A proposed operational definition. Clin. Psychol. Sci. Pract..

[16] Kabat-Zinn J. Mindfulness-Based Stress Reduction (MBSR). Authorized Curriculum Guide 2017. https://lotheijke.com/wp-content/uploads/2020/11/eight-week-mbsr-authorized-curriculum-guide-2017.pdf.

[17] Bartlett L., Martin A., Neil A.L., Memish K., Otahal P., Kilpatrick M., Sanderson K. (2019). A systematic review and meta-analysis of workplace mindfulness training randomized controlled trials. J. Occup. Health Psychol..

[18] Lomas T., Medina J.C., Ivtzan I., Rupprecht S., Eiroa-Orosa F.J. (2018). Mindfulness-based interventions in the workplace: An inclusive systematic review and meta-analysis of their impact upon wellbeing. J. Posit. Psychol..

[19] Johnson K.R., Park S., Chaudhuri S. (2020). Mindfulness training in the workplace: Exploring its scope and outcomes. Eur. J. Train. Dev..

[20] Pérez-Fuentes M.d.C., Molero Jurado M.d.M., Mercader Rubio I., Soriano Sánchez J.G., Gázquez Linares J.J. (2020). Mindfulness for Preventing Psychosocial Risks in the Workplace: A Systematic Review and Meta-Analysis. Appl. Sci..

[21] Kersemaekers W., Rupprecht S., Wittmann M., Tamdjidi C., Falke P., Donders R., Speckens A., Kohls N. (2018). A Workplace Mindfulness Intervention May Be Associated With Improved Psychological Well-Being and Productivity. A Preliminary Field Study in a Company Setting. Front. Psychol..

[22] Hülsheger U.R., Alberts H.J.E.M., Feinholdt A., Lang J.W.B. (2013). Benefits of mindfulness at work: The role of mindfulness in emotion regulation, emotional exhaustion, and job satisfaction. J. Appl. Psychol..

[23] Martínez-Borrás R., Navarrete J., Bellosta-Batalla M., Martínez-Brotóns C., Martínez-Rubio D. (2022). Changes in Salivary Immunoglobulin A, Stress, and Burnout in a Workplace Mindfulness Intervention: A Pilot Study. Int. J. Environ. Res. Public Health.

[24] El Morr C., Ritvo P., Ahmad F., Moineddin R. (2020). Effectiveness of an 8-Week Web-Based Mindfulness Virtual Community Intervention for University Students on Symptoms of Stress, Anxiety, and Depression: Randomized Controlled Trial. JMIR Ment. Health.

[25] Ma Y., She Z., Siu A.F.-Y., Zeng X., Liu X. (2018). Effectiveness of Online Mindfulness-Based Interventions on Psychological Distress and the Mediating Role of Emotion Regulation. Front. Psychol..

[26] Nadler R., Carswell J.J., Minda J.P. (2020). Online Mindfulness Training Increases Well-Being, Trait Emotional Intelligence, and Workplace Competency Ratings: A Randomized Waitlist-Controlled Trial. Front. Psychol..

[27] Querstret D., Cropley M., Fife-Schaw C. (2018). The Effects of an Online Mindfulness Intervention on Perceived Stress, Depression and Anxiety in a Non-clinical Sample: A Randomised Waitlist Control Trial. Mindfulness.

[28] Zhang Y., Xue J., Huang Y. (2020). A meta-analysis: Internet mindfulness-based interventions for stress management in the general population. Medicine.

[29] Spijkerman M.P.J., Pots W.T.M., Bohlmeijer E.T. (2016). Effectiveness of online mindfulness-based interventions in improving mental health: A review and meta-analysis of randomised controlled trials. Clin. Psychol. Rev..

[30] Sommers-Spijkerman M., Austin J., Bohlmeijer E., Pots W. (2021). New Evidence in the Booming Field of Online Mindfulness: An Updated Meta-analysis of Randomized Controlled Trials. JMIR Ment. Health.

[31] Lazarus R.S., Folkman S. (1984). Stress, Appraisal and Coping.

[32] Hobfoll S.E. (1988). The Series in Health Psychology and Behavioral Medicine. The Ecology of Stress.

[33] Ellis A., Mahoney M.J., Freeman A. (1985). Expanding the ABCs of rational-emotive therapy. Cognition and Psychotherapy.

[34] Faul F., Erdfelder E., Lang A.G., Buchner A. (2007). G*Power 3: A flexible statistical power analysis program for the social, behavioral, and biomedical sciences. Behav. Res. Methods.

[35] Bergomi C., Tschacher W., Kupper Z. (2014). Konstruktion und erste Validierung eines Fragebogens zur umfassenden Erfassung von Achtsamkeit. Diagnostica.

[36] Bergomi C., Tschacher W., Kupper Z. (2012). Measuring Mindfulness: First Steps Towards the Development of a Comprehensive Mindfulness Scale. Mindfulness.

[37] Bergomi C., Tschacher W., Kupper Z. (2012). The Assessment of Mindfulness with Self-Report Measures: Existing Scales and Open Issues. Mindfulness.

[38] Cladder-Micus M.B., Becker E.S., Spijker J., Speckens A.E.M., Vrijsen J.N. (2019). Effects of Mindfulness-Based Cognitive Therapy on a Behavioural Measure of Rumination in Patients with Chronic, Treatment-Resistant Depression. Cogn. Ther. Res..

[39] Fliege H., Rose M., Arck P., Levenstein S., Klapp B.F. (2009). PSQ. Perceived Stress Questionnaire [PSQ20—Deutsche Fassung]. Leibniz-Zentrum für Psychologische Information und Dokumentation (ZPID), Elektronisches Testarchiv.

[40] The Jamovi Project (2021). Jamovi, Version 2.2.5.0.

[41] Rasch B., Friese M., Hofmann W., Naumann E. (2021). Quantitative Methoden 2.

[42] Verma J.P. (2016). Repeated Measures Design for Empirical Researchers (1. Aufl.).

[43] Chen T., Xu M., Tu J., Wang H., Niu X. (2018). Relationship between Omnibus and Post-hoc Tests: An Investigation of performance of the F test in ANOVA. Shanghai Arch. Psychiatry.

[44] Holm K., Christman N.J. (1985). Post hoc tests following analysis of variance. Res. Nurs. Health.

[45] Vaske J.J., Beaman J., Sponarski C.C. (2016). Rethinking Internal Consistency in Cronbach’s Alpha. Leis. Sci..

[46] Lakens D. (2013). Calculating and reporting effect sizes to facilitate cumulative science: A practical primer for t-tests and ANOVAs. Front. Psychol..

[47] Rice M.E., Harris G.T. (2005). Comparing effect sizes in follow-up studies: ROC Area, Cohen’s d, and r. Law Hum. Behav..

[48] Cohen J. (1988). Statistical Power Analysis for the Behavioral Sciences.

[49] Iani L., Lauriola M., Chiesa A., Cafaro V. (2018). Associations Between Mindfulness and Emotion Regulation: The Key Role of Describing and Nonreactivity. Mindfulness.

[50] Neff K.D. (2003). The Development and Validation of a Scale to Measure Self-Compassion. Self Identity.

[51] Schulze K., Kanske P., Barnow S. (2020). Neuronale Korrelate der Emotionsregulation. Handbuch Emotionsregulation.

[52] Ellis A. (1991). The revised ABC’s of rational-emotive therapy (RET). J. Ration. -Emot. Cogn. -Behav. Ther..

[53] Flies E. (2020). Embodiment und Emotionen im Coaching 4.0.

[54] Ek S. (2015). Gender differences in health information behaviour: A Finnish population-based survey. Health Promot. Int..

[55] Verplanken B., Fisher N. (2013). Habitual Worrying and Benefits of Mindfulness. Mindfulness.

[56] Goldin P.R., Gross J.J. (2010). Effects of mindfulness-based stress reduction (MBSR) on emotion regulation in social anxiety disorder. Emotion.

[57] Hülsheger U.R. (2015). Making sure that mindfulness is promoted in organizations in the right wayand for the right goals. Ind. Organ. Psychol..

