# Supplementary material for: The Effectiveness of a Four-Week Online Mindfulness Training Course on Individual Mindfulness Skills and Personal Perception of Stress in Company Employees Working from Home
Source: Int J Environ Res Public Health. 2022 Dec 7;19(24):16422. doi: 10.3390/ijerph192416422 (PMC9778306; doi:10.3390/ijerph192416422)
Supplement: Supplementary file 1 [file ijerph-19-16422-s001.zip › ijerph-2085078-supplementary.pdf]

Supplementary File S1 - CHIME Questionnaire

Please indicate how often, in the past two weeks, you have had each of the following experiences.

	Never or Almost Never	Rarely	Not Often	Fairly Often	Often	Always or Almost Always
1. When my mood changes, I notice it right away.						
2. In the ups and downs of life, I am kind to myself.						
3. In everyday life, I notice when my negative attitudes toward a situation make things worse.						
4. It is clear to me that my evaluations of situations and people can change easily.						
5. When I am sitting or lying down, I notice the sensations in my body.						
6. I am able to smile when I notice myself seeing things as more complicated than they actually are.						
7. I am hard on myself when I make a mistake.						
8. When I experience distressing thoughts or images, I am able to feel calm soon afterward.						
9. I notice details in nature, such as colors, shapes, and textures.						
10. I break or spill things because I am not paying attention or I am thinking of something else.						
11. I experience my mistakes and difficulties without judging myself.						
12. It is easy for me to stay focused on what I am doing.						
13. When I experience distressing thoughts or images, I am able to notice them without having to react.						
14. When I talk to other people, I notice the feelings I am experiencing.						
15. When I have needlessly given myself a hard time, I can see it with humor.						
16. In difficult situations, I can pause for a moment without reacting immediately.						
17. In everyday life, I get distracted by many memories, images, or daydreams.						
18. When I ride in a car or train, I am aware of the surroundings, such as the landscape.						
19. I try to stay busy to avoid specific thoughts or feelings from coming to mind.						
20. When caught in thoughts and emotions, I am able to step back and quickly notice the thought or emotion without becoming overwhelmed.						
21. I pay attention to sensations, such as the wind in my hair or sunshine on my face.						
22. I try to distract myself when I feel unpleasant emotions.						
23. In everyday life, I realize my thoughts are not always facts.						
24. I am able to smile to myself when I notice I have made a big deal out of a small problem.						
25. I am able to notice my thoughts and feelings without getting tangled up in them.						
26. When I read, I have to reread paragraphs because I was thinking of something else.						
27. I notice sounds in my environment, such as birds chirping or cars passing.						
28. I notice my thoughts and feelings and can also step back and observe them from a distance.						
29. I clearly notice changes in my body, such as quicker or slower breathing.						
30. I do not like it when I am angry or fearful and I try to get rid of these feelings.						
31. In everyday life, I am aware that my view on things is not always based on facts.						
32. Even when I make a big mistake, I treat myself with kindness and understanding.						
33. When I experience discomfort, I try to avoid this sensation as much as possible.						
34. I am usually aware of how I am feeling at any given time.						
35. I am aware that even my strongly held opinions may change over time.						
36. I resent my own mistakes and weaknesses.						
37. I am able to notice when I unnecessarily make life more difficult for myself.						

Supplementary File S2 - Detailed statistical results on homogeneity, normality, and sphericity across all scales of the questionnaire

Results for PSQ.

		df	MS	F	p	η^2_p	implicit Hypothesis					MD	95% CI upper	lower	SE	df	t	p_{tukey}	*d*	*r*
	Time	2	0.42	3.37	0.04	0.10	T0	EXP	=	T0	CON	0.15	0.25	-0.55	0.21	29	-0.72	0.98		
	Interaction	2	0.81	6.55	0.00	0.18	T0	EXP	<	T1	EXP	-0.57	-0.26	-0.89	0.16	29	-3.59	0.01	1.33	0.55
PSQ	Residual	58	0.12				T0	CON	=	T1	CON	0.07	0.42	-0.27	0.18	29	0.41	1.00		
	Group	1	3.44	4.26	0.05	0.13	T1	EXP	≠	T1	CON	0.50	0.82	0.18	0.16	29	3.05	0.05	1.13	0.49
	Residual	29	0.81				T1	EXP	=	T2	EXP	0.08	0.36	-0.20	0.14	29	0.56	0.99		
							T1	CON	=	T2	CON	-0.09	0.21	-0.40	0.16	29	-0.60	0.99		
							T2	EXP	≠	T2	CON	0.32	0.74	-0.09	0.21	29	1.52	0.66		
		df	MS	F	p	η^2_p	implicit Hypothesis					MD	95% CI upper	lower	SE	df	t	p_{tukey}	*d*	*r*
	Time	2	0.66	3.68	0.03	0.11	T0	EXP	=	T0	CON	0.04	0.50	-0.41	0.23	29	0.19	1.00		
	Interaction	2	0.86	4.75	0.01	0.14	T0	EXP	<	T1	EXP	-0.45	-0.17	-0.72	0.14	29	-3.18	0.04	1.18	0.51
PSQ-JOY	Residual	58	0.18				T0	CON	=	T1	CON	0.20	0.50	-0.10	0.16	29	1.29	0.79		
	Group	1	1.16	2.24	0.15	0.07	T1	EXP	≠	T1	CON	0.69	1.05	0.33	0.18	29	3.79	0.01	1.41	0.58
	Residual	29	0.52				T1	EXP	=	T2	EXP	0.02	0.24	-0.19	0.11	29	0.22	1.00		
							T1	CON	=	T2	CON	-0.24	-0.01	-0.48	0.12	29	-2.04	0.35		
							T2	EXP	≠	T2	CON	0.42	0.87	-0.02	0.23	29	1.88	0.43		
		df	MS	F	p	η^2_p	implicit Hypothesis					MD	95% CI upper	lower	SE	df	t	p_{tukey}	*d*	*r*
	Time	2	0.28	1.06	0.34	0.04	T0	EXP	=	T0	CON	-0.01	0.39	-0.41	0.20	29	-0.05	1.00		
	Interaction	2	0.42	1.59	0.22	0.05	T0	EXP	<	T1	EXP	-0.24	0.12	-0.59	0.18	29	-1.31	0.78		
PSQ-SORG	Residual	47	0.26				T0	CON	=	T1	CON	0.16	0.55	-0.23	0.20	29	0.79	0.97		
	Group	1	1.22	1.96	0.17	0.06	T1	EXP	≠	T1	CON	0.38	0.77	0.00	0.20	29	1.94	0.40		
	Residual	29	0.62				T1	EXP	=	T2	EXP	-0.09	0.24	-0.43	0.17	29	-0.54	0.99		
							T1	CON	=	T2	CON	-0.16	0.22	-0.53	0.19	29	-0.82	0.96		
							T2	EXP	≠	T2	CON	0.32	0.78	-0.15	0.24	29	1.34	0.76		
		df	MS	F	p	η^2_p	implicit Hypothesis					MD	95% CI upper	lower	SE	df	t	p_{tukey}	*d*	*r*
	Time	2	1.02	5.14	0.01	0.15	T0	EXP	=	T0	CON	-0.05	0.35	-0.44	0.20	29	-0.22	1.00		
	Interaction	2	1.38	6.94	0.00	0.19	T0	EXP	<	T1	EXP	-0.65	-0.34	-0.96	0.16	29	-4.06	0.00	1.51	0.60
PSQ-ANSP	Residual	58	0.20				T0	CON	=	T1	CON	0.10	0.44	-0.24	0.18	29	0.57	0.99		
	Group	1	4.53	8.26	0.01	0.22	T1	EXP	≠	T1	CON	0.70	1.04	0.36	0.17	29	4.06	0.00	1.51	0.60
	Residual	29	0.55				T1	EXP	=	T2	EXP	-0.06	0.26	-0.37	0.16	29	-0.36	1.00		
							T1	CON	=	T2	CON	-0.09	0.26	-0.43	0.18	29	-0.48	1.00		
							T2	EXP	≠	T2	CON	0.67	1.12	0.23	0.23	29	2.94	0.06		
		df	MS	F	p	η^2_p	implicit Hypothesis					MD	95% CI upper	lower	SE	df	t	p_{tukey}	*d*	*r*
	Time	2.00	1.63	10.88	< .001	0.27	T0	EXP	=	T0	CON	-0.31	0.10	-0.71	0.21	29	-1.50	0.67		
	Interaction	2.00	0.70	4.67	0.01	0.14	T0	EXP	<	T1	EXP	-0.74	-0.50	-0.98	0.12	29	-6.15	< .001	2.28	0.75
PSQ-ANFO	Residual	58	0.15				T0	CON	=	T1	CON	-0.17	0.09	-0.43	0.13	29	-1.29	0.79		
	Group	1	0.03	0.04	0.84	0.00	T1	EXP	≠	T1	CON	0.26	0.61	-0.09	0.18	29	1.46	0.69		
	Residual	29	0.65				T1	EXP	=	T2	EXP	0.22	0.51	-0.06	0.14	29	1.55	0.64		
							T1	CON	=	T2	CON	0.11	0.43	-0.20	0.16	29	0.72	0.98		
							T2	EXP	≠	T2	CON	0.15	0.59	-0.28	0.22	29	0.69	0.98		

df = degrees of freedom, MS = mean square, F = F-Value, p = level of significance, η2p = partial eta-squared, MD = mean difference, CI = confidence interval, SE = standard Error, t = t-value for post-hoc comparison, p = level of significance, d = Cohen's Delta, r = Pearson Product Moment.

Results for CH

		df	MS	F	p	η^2_p	implicit Hypothesis					MD	95% CI upper	95% CI lower	SE	df	t	p_{tukey}	*d*	*r*
	Time	2.00	0.58	6.28	0.00	0.18	T0	EXP	=	T0	CON	0.28	0.72	-0.15	0.22	29	1.27	0.80		
	Interaction	2.00	0.98	10.73	< .001	0.27	T0	EXP	<	T1	EXP	-0.49	-0.29	-0.69	0.10	29	-4.80	< .001	1.78	0.67
CH	*Residual*	58	0.09				T0	CON	=	T1	CON	0.08	0.30	-0.14	0.11	29	0.68	0.98		
	Group	1	11.05	10.40	0.00	0.26	T1	EXP	≠	T1	CON	0.85	1.31	0.40	0.23	29	3.68	0.01	1.37	0.56
	Residual	29	1.06				T1	EXP	=	T2	EXP	-0.10	0.09	-0.29	0.10	29	-1.00	0.91		
							T1	CON	=	T2	CON	0.00	0.20	-0.21	0.11	29	-0.04	1.00		
							T2	EXP	≠	T2	CON	0.94	1.42	0.47	0.24	29	3.89	0.01	1.45	0.59

		df	MS	F	p	η^2_p	implicit Hypothesis					MD	95% CI upper	95% CI lower	SE	df	t	p_{tukey}	*d*	*r*
	Time	2.00	1.67	6.03	0.00	0.17	T0	EXP	=	T0	CON	0.64	1.34	-0.06	0.36	29	1.78	0.49		
	Interaction	2.00	0.96	3.48	0.04	0.11	T0	EXP	<	T1	EXP	-0.59	-0.21	-0.97	0.19	29	-3.05	0.05	1.13	0.49
CH AMH	*Residual*	58	0.28				T0	CON	=	T1	CON	-0.10	0.32	-0.52	0.21	29	-0.47	1.00		
	Group	1	24.35	9.55	0.00	0.25	T1	EXP	≠	T1	CON	1.12	1.84	0.41	0.37	29	3.07	0.05	1.14	0.50
	Residual	29	2.55				T1	EXP	=	T2	EXP	-0.20	0.19	-0.59	0.20	29	-1.02	0.91		
							T1	CON	=	T2	CON	0.00	0.00	0.00	0.22	29	< 0.01	1.00		
							T2	EXP	≠	T2	CON	1.32	2.06	0.58	0.38	29	3.51	0.02	1.30	0.55

		df	MS	F	p	η^2_p	implicit Hypothesis					MD	95% CI upper	95% CI lower	SE	df	t	p_{tukey}	*d*	*r*
	Time	2.00	0.23	0.61	0.55	0.02	T0	EXP	=	T0	CON	-0.55	0.08	-1.19	0.32	29	-1.70	0.54		
	Interaction	2.00	2.51	6.68	0.00	0.19	T0	EXP	<	T1	EXP	-0.72	-0.27	-1.17	0.23	29	-3.17	0.04	1.18	0.51
CH BHG	*Residual*	58	0.38				T0	CON	=	T1	CON	0.41	0.90	-0.08	0.25	29	1.64	0.58		
	Group	1	0.09	0.06	0.81	0.00	T1	EXP	≠	T1	CON	0.58	1.13	0.03	0.28	29	2.06	0.34		
	Residual	29	1.50				T1	EXP	=	T2	EXP	0.22	0.60	-0.16	0.20	29	1.13	0.86		
							T1	CON	=	T2	CON	-0.20	0.23	-0.62	0.22	29	-0.91	0.94		
							T2	EXP	≠	T2	CON	0.16	0.81	-0.49	0.33	29	0.49	1.00		

		df	MS	F	p	η^2_p	implicit Hypothesis					MD	95% CI upper	95% CI lower	SE	df	t	p_{tukey}	*d*	*r*
	Time	2.00	0.62	2.91	0.06	0.09	T0	EXP	=	T0	CON	0.59	1.11	0.07	0.27	29	2.22	0.26		
	Interaction	2.00	0.53	2.47	0.09	0.08	T0	EXP	<	T1	EXP	-0.47	-0.13	-0.82	0.18	29	-2.67	0.11		
CH EV	*Residual*	58	0.21				T0	CON	=	T1	CON	0.03	0.41	-0.35	0.19	29	0.15	1.00		
	Group	1	18.13	11.30	0.00	0.28	T1	EXP	≠	T1	CON	1.09	1.70	0.48	0.31	29	3.49	0.02	1.30	0.54
	Residual	29	1.60				T1	EXP	=	T2	EXP	0.01	0.27	-0.25	0.13	29	0.09	1.00		
							T1	CON	=	T2	CON	-0.10	0.19	-0.39	0.15	29	-0.68	0.98		
							T2	EXP	≠	T2	CON	0.98	1.59	0.37	0.31	29	3.16	0.04	1.17	0.51

		df	MS	F	p	η^2_p	implicit Hypothesis					MD	95% CI upper	95% CI lower	SE	df	t	p_{tukey}	*d*	*r*
	Time	2.00	0.92	2.59	0.08	0.08	T0	EXP	=	T0	CON	0.47	1.25	-0.31	0.40	29	1.18	0.84		
	Interaction	2.00	0.57	1.61	0.21	0.05	T0	EXP	<	T1	EXP	-0.46	0.00	-0.91	0.23	29	-1.96	0.39		
CH GÄE	*Residual*	58	0.36				T0	CON	=	T1	CON	-0.09	0.41	-0.59	0.26	29	-0.35	1.00		
	Group	1	13.65	6.02	0.02	0.17	T1	EXP	≠	T1	CON	0.84	1.44	0.23	0.31	29	2.71	0.10		
	Residual	29	2.27				T1	EXP	=	T2	EXP	-0.13	0.22	-0.48	0.18	29	-0.74	0.98		
							T1	CON	=	T2	CON	0.04	0.42	-0.35	0.20	29	0.18	1.00		
							T2	EXP	≠	T2	CON	1.00	1.72	0.29	0.37	29	2.74	0.10		

		df	MS	F	p	η^2_p	implicit Hypothesis					MD	95% CI upper	95% CI lower	SE	df	t	p_{tukey}	*d*	*r*
	Time	1.67	0.87	2.40	0.11	0.08	T0	EXP	=	T0	CON	0.23	0.98	-0.52	0.38	29	0.60	0.99		
	Interaction	1.67	1.21	3.33	0.05	0.10	T0	EXP	<	T1	EXP	-0.60	-0.17	-1.03	0.22	29	-2.71	0.10		
CH GIE	*Residual*	48	0.36				T0	CON	=	T1	CON	0.03	0.51	-0.45	0.24	29	0.12	1.00		
	Group	1	9.62	5.46	0.03	0.16	T1	EXP	≠	T1	CON	0.86	1.43	0.29	0.29	29	2.94	0.06		
	Residual	29	1.76				T1	EXP	=	T2	EXP	0.05	0.34	-0.24	0.15	29	0.32	1.00		
							T1	CON	=	T2	CON	0.04	0.36	-0.27	0.16	29	0.27	1.00		
							T2	EXP	≠	T2	CON	0.85	1.40	0.31	0.28	29	3.07	0.05	1.14	0.49

		df	MS	F	p	η^2_p	implicit Hypothesis					MD	95% CI upper	95% CI lower	SE	df	t	p_{tukey}	*d*	*r*
	Time	2.00	0.73	2.74	0.07	0.09	T0	EXP	=	T0	CON	0.36	0.95	-0.24	0.30	29	1.18	0.84		
	Interaction	2.00	2.21	8.28	< .001	0.22	T0	EXP	<	T1	EXP	-0.58	-0.21	-0.94	0.19	29	-3.12	0.04	1.16	0.50
CH NRH	*Residual*	58	0.27				T0	CON	=	T1	CON	0.14	0.54	-0.26	0.21	29	0.70	0.98		
	Group	1	20.58	13.00	0.00	0.31	T1	EXP	≠	T1	CON	1.08	1.69	0.46	0.31	29	3.44	0.02	1.28	0.54
	Residual	29	1.58				T1	EXP	=	T2	EXP	-0.25	0.12	-0.61	0.19	29	-1.31	0.78		
							T1	CON	=	T2	CON	0.08	0.49	-0.32	0.21	29	0.40	1.00		
							T2	EXP	≠	T2	CON	1.40	1.98	0.83	0.30	29	4.77	< .001	1.77	0.66
		df	MS	F	p	η^2_p	implicit Hypothesis					MD	95% CI upper	95% CI lower	SE	df	t	p_{tukey}	*d*	*r*
	Time	2.00	1.06	3.64	0.03	0.11	T0	EXP	=	T0	CON	0.00	0.49	-0.48	0.25	29	0.01	1.00		
	Interaction	2.00	1.03	3.55	0.04	0.11	T0	EXP	<	T1	EXP	-0.71	-0.38	-1.03	0.17	29	-4.27	0.00	1.59	0.62
CH OHV	*Residual*	58	0.29				T0	CON	=	T1	CON	0.00	0.00	0.00	0.18	29	< 0.00	1.00		
	Group	1	3.95	3.38	0.08	0.11	T1	EXP	≠	T1	CON	0.71	1.22	0.20	0.26	29	2.73	0.10	1.01	0.45
	Residual	29	1.17				T1	EXP	=	T2	EXP	0.16	0.60	-0.27	0.22	29	0.75	0.97		
							T1	CON	=	T2	CON	-0.01	0.46	-0.49	0.24	29	-0.06	1.00		
							T2	EXP	≠	T2	CON	0.53	1.15	-0.09	0.32	29	1.69	0.55		
		df	MS	F	p	η^2_p	implicit Hypothesis					MD	95% CI upper	95% CI lower	SE	df	t	p_{tukey}	*d*	*r*
	Time	2.00	0.05	0.14	0.87	0.01	T0	EXP	=	T0	CON	0.53	1.08	-0.02	0.28	29	1.90	0.42		
	Interaction	2.00	1.21	3.55	0.04	0.11	T0	EXP	<	T1	EXP	-0.31	0.15	-0.77	0.23	29	-1.32	0.77		
CH R	*Residual*	58	0.34				T0	CON	=	T1	CON	0.27	0.77	-0.24	0.26	29	1.04	0.90		
	Group	1	21.93	15.30	< .001	0.35	T1	EXP	≠	T1	CON	1.11	1.72	0.49	0.31	29	3.54	0.02	1.31	0.55
	Residual	29	1.43				T1	EXP	=	T2	EXP	-0.15	0.22	-0.51	0.19	29	-0.80	0.97		
							T1	CON	=	T2	CON	0.04	0.44	-0.36	0.20	29	0.18	1.00		
							T2	EXP	≠	T2	CON	1.29	1.91	0.67	0.31	29	4.11	0.00	1.52	0.61

df = degrees of freedom, MS = mean square, F = F-Value, p = level of significance, η2p = partial eta-squared, MD = mean difference, CI = confidence interval, SE = standard Error, t = t-value for post-hoc comparison, p = level of significance, d = Cohen's Delta, r = Pearson Product Moment.
